# Ischemic Stroke Secondary to a Stab Wound to the Neck in a Young Adult

**DOI:** 10.1155/2022/9365947

**Published:** 2022-10-18

**Authors:** Mateo Zuluaga-Gómez, Daniela Giraldo-Campillo, Daniel González-Arroyave, Carlos M. Ardila

**Affiliations:** ^1^Hospital San Vicente Fundación, Rionegro, Colombia; ^2^Profesor Universidad Bolivariana, Medellín, Colombia; ^3^Universidad de Antioquia, Medellín, Colombia

## Abstract

Vertebral artery dissection is a common cause of stroke in young adults without predisposing risk factors for cerebrovascular disease. We describe the case of a 28-year-old patient who presented with an ischemic stroke secondary to a stab wound to the neck that affected the vertebral artery. A physical examination revealed neurological deterioration (Glasgow 8/15), a sutured neck wound, no palpable hematoma, no thrills, and no active bleeding. A computed tomography angiography revealed a left vertebral artery arteriovenous fistula with a component of a pseudoaneurysm, for which a neurointerventional consultation was carried out. Due to neurological compromise, the airway was secured, and because the case involved a posterior fossa infarction with compression of the fourth ventricle and obstructive secondary hydrocephalus, an external ventricular shunt was inserted by neurosurgery. A fistula occlusion was performed with five Axium coils and a vial of Squid 12; the vertebral artery was catheterized, and a craniotomy was performed to manage hydrocephalus with a 12-mm H_2_O collecting system. The patient was discharged on the tenth day after admission with sequelae of left hemiparesis (predominantly brachial) and no other deficits. There was no hemorrhagic transformation on the control computed tomography scans and no further complications.

## 1. Introduction

Vertebral artery injuries can be spontaneous or caused by trauma. Traumatic injuries are frequently caused by blunt head and neck injuries but can also be caused by penetrating trauma [1]. Vertebral artery partial dissection is a common cause of stroke in young adults without predisposing risk factors for cerebrovascular disease [2]. Initial signs of vertebral artery dissection are usually head and neck pain [3]; however, patients may be asymptomatic [1]. The vertebral arteries perfuse the posterior fossa, and dissection causes apoplexy symptoms consistent with a posterior circulation deficit. Difficulty speaking, swallowing, and maintaining balance may also be observed in addition to the loss of coordination and/or changes in vision [1].

Most injuries to the vertebral arteries are due to blunt trauma from motor vehicle accidents, and cases related to sports, trampoline use, sexual intercourse, yoga, scuba diving, and chiropractic neck manipulation have been reported [1]. However, a few cases have been reported in association with stab wounds and strokes. This report describes an ischemic stroke secondary to a stab wound to the neck in a young adult with no history of cerebrovascular disease.

## 2. Case Report

A 28-year-old male patient with a traumatic wound in zone II of the neck produced after being stabbed during a fight was initially taken to a lower-complexity hospital, where he was discharged because no serious tissue injuries were found. Computed tomography angiography was not performed in this hospital because this institution does not have this resource; however, initial stabilization was implemented, the wound was sutured, analgesics were prescribed, and a tetanus toxoid vaccine was administered. However, 2 days later, the patient consulted a higher-level hospital for episodes of hematemesis and syncope.

**Figure 1:**
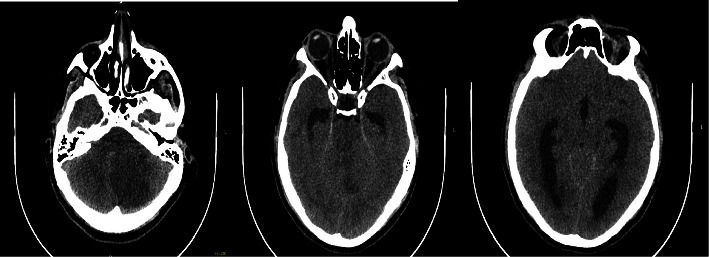
Simple skull tomography. (a). Infarction in the left cerebellar hemisphere. (b). Collapse of the fourth ventricle and hydrocephalus. (c). Dilation of the third ventricle and lateral ventricles.

**Figure 2:**
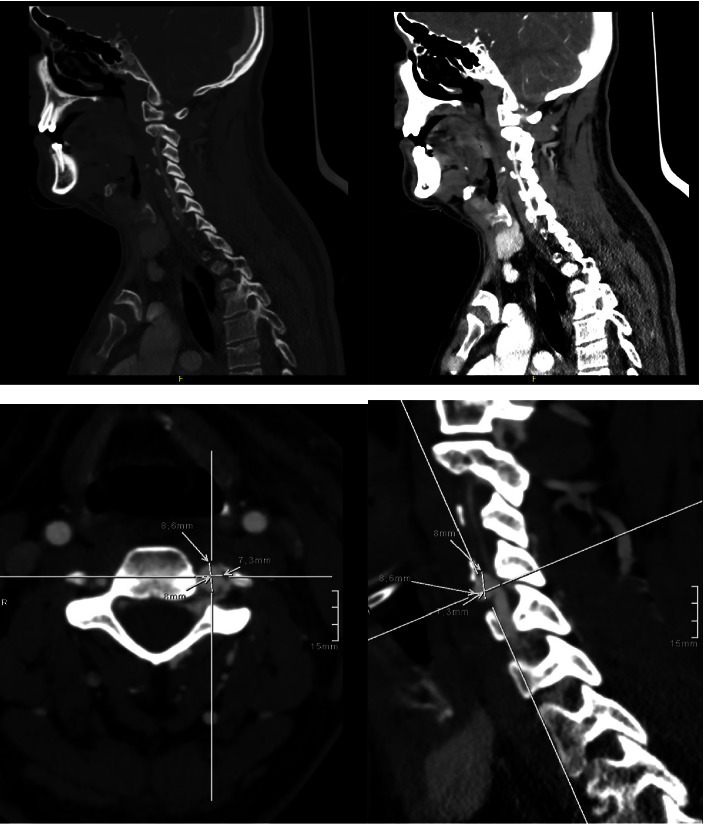
Angiography of the head and neck vessels. A pseudoaneurysm of the left vertebral artery is observed at the level of C4 with an associated arteriovenous fistula and without signs of active bleeding. The pseudoaneurysm measures 8.5 mm anteroposterior diameter, 7.3 mm transverse diameter, and 8 mm craniocaudal diameter. Hyperdensity of adjacent venous structures is observed, and the venous phase is better visualized after configuring an arteriovenous fistula with a filiform filling of the distal vertebral artery.

**Figure 3:**
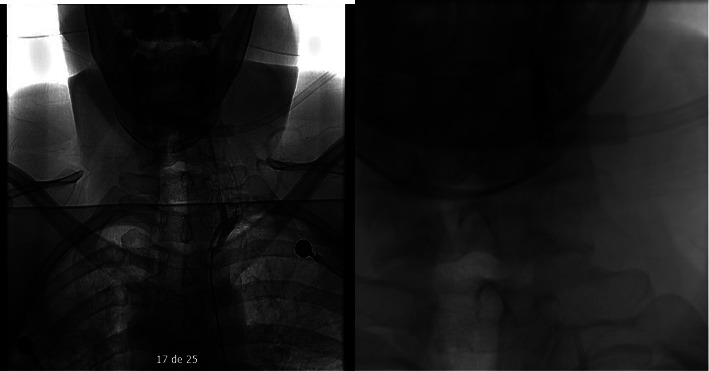
Panangiography depicting vertebral artery occlusion. The fistula is occluded with five Axium coils and a vial of Squid 12.

Upon hospital admission, he presented the following vital signs: blood pressure 130/78 mmHg, heart rate 100 beats per minute, and oxygen saturation 100% on room air. A physical examination revealed neurological deterioration (Glasgow 8/15), a sutured neck wound, no palpable hematoma, no thrills, no active bleeding, and no other findings. An emergent simple skull computed tomography (CT) was performed, which indicated cerebellar stroke and obstructing hydrocephalus (Figures 1 and 2). An emergent head and neck angiotomography indicated a compromise of the vertebral artery with a pseudoaneurysm (Figure 3). An emergent panangiography was scheduled in consultation with a neurointerventional service. CT angiography revealed a compromise of the left vertebral artery with an appearance suggestive of a pseudoaneurysm, for which a neurointerventional consultation was carried out.

Due to neurological compromise, we decided to secure the airway, and because this case involved a posterior fossa infarction with compression of the fourth ventricle and secondary obstructive hydrocephalus, the insertion of an external ventricular shunt was performed by neurosurgery. Subsequently, neurointerventional fistula occlusion was performed with five coils and a vial of Squid 12; the vertebral artery was catheterized, and a craniotomy was performed by neurosurgery to manage hydrocephalus with a 12-mm H_2_O collecting system.

**Figure 4:**
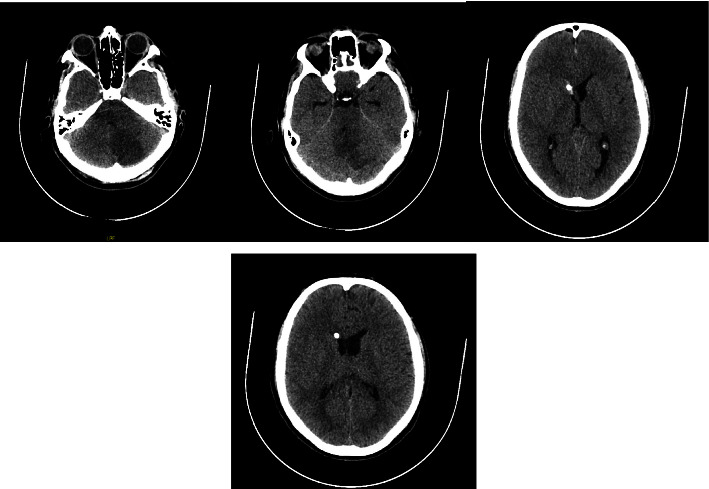
Simple control computed tomography of the skull. (a). Established infarction in the left cerebellar hemisphere. (b). Ventricular and cisternal systems with normal shape, size, and position. (c). A ventricular bypass catheter enters through the right frontal region, with the distal end in the frontal horn of the right lateral ventricle. (d). Decrease in size of the right ventricular system compared with the contralateral side.

The patient was then transferred to the intensive care unit.  Figure 4 presents a simple skull control tomography showing the normal shape, size, and position of the ventricular and cisternal systems and a decrease in the size of the right ventricular system compared with the contralateral side. The external ventricular shunt was removed after clinical and CT improvement at 72 hours.

The patient was discharged on the tenth day after admission with sequelae of left hemiparesis (predominantly brachial) and no other deficits. There was no hemorrhagic transformation on the control CT scans and no further complications.

The patient's sister signed the informed consent form for publication purposes. The report on this case was endorsed by the Bioethics Committee of the Hospital San Vicente Fundación.

## 3. Discussion

Traumatic vertebral artery injury is a relatively rare condition, reported in less than 1% of trauma admissions. However, a recent increase in incidence has been described, probably due to greater awareness and detection of this condition in high-risk patients [1, 4, 5]. Although the literature reports cases of traumatic injury to the vertebral artery by knives [5–9], there are few cases involving ischemic stroke secondary to a stab wound to the neck that affects the vertebral artery. Similar to the present case, Park et al. described a case of cerebellar infarction produced by vertebral artery injury from a stab wound that separated the vessel between the transverse processes of C3 and C4 with a hypoplastic contralateral vertebral artery. The patient suffered an infarction of the cerebellum due to insufficient preservation of blood flow in the posterior inferior cerebellar artery [10].

The neurologic consequences of vertebral artery injury are due to cerebral ischemia from thromboembolism, hypoperfusion, hemorrhage, or a combination of these. Generally, vertebral artery injury is caused by an intimal tear, generating platelet aggregation and thrombus formation that can cause local occlusion of the vessel. However, more often, the clot will embolize the cerebral circulation, causing a stroke [1, 4].

As observed in the present case, partial dissection of the vertebral artery can also cause a pseudoaneurysm, which can become a source of emboli or hemorrhage [1, 11] Moreover, the risk of stroke is highest in the first 2 weeks after partial dissection [1].

CT angiography (CTA) is the most reliable noninvasive neurovascular imaging modality and is the initial test of choice for patients with suspected cerebral artery injury. However, digital subtraction arteriography (DSA) has been indicated as the gold standard, although it is more invasive, less available, and requires more contrast than angiography. Consequently, CTA has replaced DSA [1, 5, 7, 11].

Symptomatic patients may be treated with anticoagulation over antiplatelet therapy, depending on the risk of bleeding and the location and extent of the lesion. As in the present case, endovascular therapy and surgical repair are reserved for patients with high-grade injuries [1, 7, 9, 11]; it is also recommended for patients with contraindications to anticoagulant or antiplatelet therapy who are at high risk of progression [1].

The prognosis for patients with a vertebral artery injury reflects a 24% stroke rate and an 8% mortality rate; between 5% and 25% of patients have poor neurological outcomes [5]. Poor outcomes are associated with older age, arterial occlusion, and increased stroke severity at the time of diagnosis [4].

## Data Availability

The clinical data utilized in this report are described in this article.
